# The role and possible mechanism of intestinal fungi in the progression of chronic liver diseases

**DOI:** 10.1038/s41522-026-00961-5

**Published:** 2026-03-10

**Authors:** Yirui Hu, Ye Yang, Shuyan Wang, Huikuan Chu

**Affiliations:** Division of Gastroenterology, Union Hospital, Tongji Medical College, Huazhong University of Science and Technology, Wuhan, Hubei China

**Keywords:** Diseases, Gastroenterology, Microbiology

## Abstract

Chronic liver disease (CLD) causes 2 million annual deaths (4% of all global deaths). While gut bacteria are widely studied, intestinal fungi remain largely overlooked despite their critical roles in maintaining microecological homeostasis. This review summarizes fungal characteristics in alcohol-related liver disease, metabolic dysfunction-associated steatotic liver disease, primary sclerosing cholangitis, and cirrhosis, analyzing roles of fungi and their metabolites. Targeting the gut fungal community may offer therapeutic strategies for CLD.

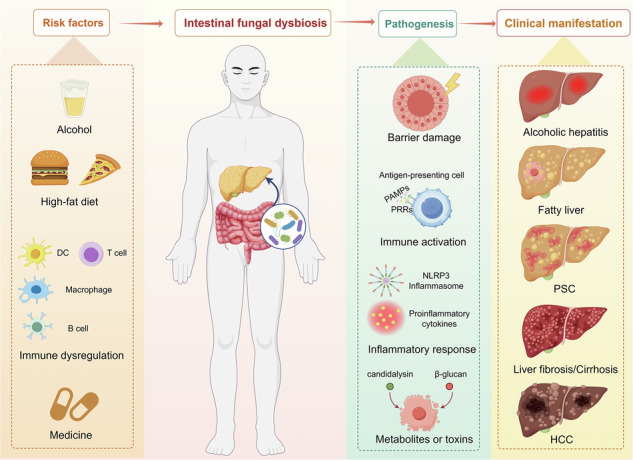

## Introduction

Worldwide, approximately 2 million people die from liver diseases annually, with one million attributable to complications of cirrhosis^1^. Chronic liver diseases (CLDs), including viral hepatitis, alcohol-related liver disease (ALD), metabolic dysfunction-associated steatotic liver disease (MASLD), autoimmune liver diseases, cirrhosis, and liver cancer, pose a major threat to global public health^2–4^. The progression of CLDs is frequently accompanied by severe complications such as infections, gastrointestinal bleeding, and hepatic encephalopathy, which contribute to their high mortality. Among these, infections are the most common complication, and most pathogenic organisms originate from the gut microbiota. Accumulating evidence underscores the crucial role of gut microbiota in the development and progression of CLDs^5,6^. Although gut bacteria have been extensively studied, intestinal fungi remain relatively under-explored, despite accounting for about 0.1% of the gut microbiota^7^. Nevertheless, fungi exhibit larger genomes, greater genetic variability, and distinct immune and metabolic functions compared to bacteria, making them potentially significant in human health and disease^8,9^.

The interaction between intestinal fungi and the liver is bidirectional^10^. Beneficial fungi can protect against pathogens and modulate immune responses, while liver immune cells may respond to fungal signals to maintain homeostasis^11^. However, approximately 70% of hepatic blood supply comes from the intestine via the portal vein, allowing fungi or their metabolites to translocate across the gut barrier and potentially exacerbate liver disease^10,12^. Currently, the fungal community is increasingly recognized as an important factor in the normal physiological functions of the liver and the occurrence and progression of CLDs^10,13^. Fungal dysbiosis in CLDs is marked by reduced alpha diversity, with depletion of commensal fungi (e.g., *Debaryomyces*, *Saccharomyces*) and overgrowth of pathobionts (e.g., *Candida*, *Mucor*). In ALD, *Candida* enrichment and *Saccharomyces* reduction are well-documented, while MASLD shows elevated *Candida*/*Mucor* and diminished *Malassezia*^14–16^. Key fungi drive CLD progression: *Candida albicans* (*C. albicans*)-derived candidalysin directly triggers hepatocyte apoptosis in ALD, and *Mucor* spp. abundance correlates with MASLD severity and fibrosis^15,17^. These findings underscore the critical role of mycobiota in CLDs.

Fungal contributions to CLDs involve gut barrier disruption, immune activation via pattern recognition receptors (PRRs), and toxin/metabolite-mediated hepatotoxicity (e.g., candidalysin)^12,17–19^. This is a narrative review that summarizes evidence on gut mycobiota alterations in CLDs and their mechanistic links to disease progression, offering novel perspectives for therapeutic targeting. The relevant studies that have been published so far are basically included in this article, and some repetitive studies (such as those that only change the sample size without altering the conclusion) have been excluded. Furthermore, we have elaborated on animal research and human clinical research separately and have not made a comparison.

## Overview of intestinal fungi

### Composition and classification of intestinal fungi

The fungal community, although it accounts for a small proportion of the entire intestinal microbiome, plays a non-negligible role in human health and disease^20^. In recent years, with the development and wide application of high-throughput sequencing technology, researchers have gained a deeper understanding of the diversity, characteristics, and distribution of the intestinal fungal community. In the intestinal fungal community of healthy humans, Ascomycota, Basidiomycota, Mucoromycota, and Chytridiomycota are the four main phyla, while *Candida, Saccharomyces*, and *Cladosporium* are the most abundant genera^21,22^. Among them, Ascomycota is the most abundant phylum. The most abundant genera in fecal samples include *C. albicans, S. cerevisiae, Penicillium, Aspergillus, Cryptococcus neoformans, Malassezia, Cladosporium, Debaryomyces*, and *Mucor*. The most commonly detected fungal species are *S. cerevisiae*, the *Malassezia restricta (M. restricta), and C. albicans*^10^.

### Characteristics and distribution of intestinal fungi

The distribution of intestinal fungi differs considerably across gastrointestinal regions from the oral cavity to the colon^23^. Generally, fungal abundance is lower in the small intestine compared to the large intestine, due to factors such as higher pH, elevated oxygen content, and high bile acid (BA) concentrations that hinder colonization^24,25^. In addition to pH, oxygen, and substrate availability, intestinal motility, chyme flow rate, local immune responses, and inflammation also collectively shape fungal distribution. The rapid flow and strong peristalsis in the small intestine favor fast-growing or mucosa-adhering fungi, including *C. albicans, S. cerevisiae*, and *Candida fibrospora*^26^.

The intestinal mycobiota also varies between the lumen and mucosal surfaces, with the microhabitat (lumen vs. mucosa) being a stronger determinant of community composition than longitudinal position along the gut^26,27^. Mucosal fungi are predominantly Ascomycota, especially *Candida* and *Saccharomyces*, whereas luminal communities exhibit higher alpha diversity and include transient environmental genera such as *Cladosporium* and *Aspergillus*^26^. Host factors—such as genetics, age, comorbidities, medication use (e.g., antibiotics), immune status, and lifestyle (hygiene, socioeconomic status, occupation)—also influence fungal composition^28^. Additionally, environmental factors like geography, urbanization, and diet play important roles, among which urbanization shows the strongest correlation with reduced fungal diversity, followed by geographic, dietary, and ethnic factors^12,29^.

Given the influence of these confounding variables, caution is necessary when interpreting studies on fungal alterations in CLDs. For instance, patients with advanced liver diseases frequently use antibiotics, which significantly alter the fungal community^30^. This may mask the true disease-related fungal characteristics. Beyond medication, diet is the main regulator of the intestinal microbiota. A western diet high in fat or sugar directly promotes the spread of specific fungi such as *Candida*^31^. Furthermore, factors such as geographical location, lifestyle, hygiene habits, and environmental exposure lead to significant variations in the microbiome among individuals and populations. This also means that the findings of one cohort may not be directly applicable to other cohorts. These various confounding factors are quite common in case-control studies and may lead to biased results. Therefore, future case‑control studies are recommended to incorporate detailed dietary surveys, medication records, and demographic data, etc. By controlling these key variables, it will be beneficial for analyzing the role of the gut microbiota in disease progression.

### Research methods of intestinal fungi

The research methods of intestinal fungi mainly include culture methods, amplicon sequencing, metagenomic sequencing, and metabolomics, etc. Although culture methods allow for the isolation of live strains, they have low sensitivity and require long incubation times. Amplicon sequencing is more efficient, accurate, and sensitive than culture methods. Metagenomic sequencing directly sequences the total DNA of the sample and can simultaneously study bacteria, fungi, and viruses. It has become an important means for studying intestinal microbiota at present. Metabolomics can directly reflect the fungal activity by detecting their metabolites.

However, it must be acknowledged that the heterogeneity of the various research methods poses significant challenges to the comparability of the data and the interpretation of fungal changes. For instance, the selection of the ITS1 versus ITS2 region for amplicon sequencing can lead to differential amplification efficiencies and taxonomic biases, with ITS1 primers often favoring Ascomycota and ITS2 better capturing Basidiomycota. This can result in divergent community structures reported for the same disease across different studies. Similarly, although quantitative PCR (qPCR) is highly sensitive to specific taxa, its accuracy is entirely dependent on the specificity of the primer/probe set. Furthermore, the detection of fungi by culture methods depends on growth conditions and may miss uncultivable or fastidious species, while metagenomic sequencing may underrepresent fungi due to their low abundance relative to bacteria. Consequently, the research results based on different core technologies may not be directly compared. This methodological heterogeneity, compounded by variations in DNA extraction protocols, sequencing platforms, and bioinformatic pipelines (including reference databases), contributes to the inconsistent reports of key fungal signatures in CLDs.

In summary, each approach has distinct advantages and limitations, and their integrated application allows for a more comprehensive understanding of the intestinal fungal community. Therefore, future research should focus on standardizing the methods, which is of vital importance for enhancing the reproducibility, comparability, and clinical applicability of gut microbiota studies.

### The importance of intestinal fungi in liver disease research

The intestine and liver are intimately connected both structurally and functionally through the gut-liver axis, enabling bidirectional communication of microbial metabolites, nutrients, and signaling molecules through the portal vein and biliary tract^32,33^. Intestinal fungi contribute to liver disease pathogenesis through direct colonization or via their metabolites and secreted factors, which exert nutritional, anti-inflammatory, and antibacterial effects^20^. In return, the liver modulates the intestinal microbiota by secreting BAs, immunoglobulin A, and antimicrobial molecules into the intestine^34^. Fungal metabolites help maintain gut-liver homeostasis, and dysbiosis is closely linked to the progression of CLDs^35^. Although the mechanisms are not fully elucidated, disruptions in this balance contribute to liver pathology^26^. Understanding the roles of intestinal fungi is therefore crucial for developing novel prevention and treatment strategies for liver diseases.

## The clinical association between intestinal fungi and CLDs

### Alcohol-related liver disease (ALD)

ALD, caused by chronic excessive alcohol consumption, represents a major global cause of liver-related morbidity and mortality, characterized by rapid progression and frequent late-stage diagnosis^36–39^. Alcohol metabolism generates toxic metabolites, which cause tissue and organ damage through an inflammatory cascade reaction involving various cytokines, chemokines, and reactive oxygen species, and its hepatotoxicity has been confirmed^36,40^. Beyond direct alcohol injury, gut microbiota dysbiosis plays a key pathogenic role. More than half of patients with alcoholism exhibit impaired intestinal barrier function and microbial imbalance^40,41^. Recent evidence indicates that alcohol-induced dysbiosis and increased intestinal permeability promote translocation of bacterial and fungal products—such as lipopolysaccharide (LPS) and β-glucan—to the liver, exacerbating inflammatory liver damage^17,40,42^. These findings underscore a significant role for intestinal fungi in the pathogenesis of ALD.

### Changes in the fungal communities of ALD patients

ALD patients show significant changes in the fungal communities composition (Table 1). At the phylum level, studies report a significant increase in Basidiomycota and a decrease in Ascomycota^16^. *C. albicans* is frequently identified as the most abundant species and is strongly associated with ALD pathogenesis^14,43^. However, some studies report a shift in dominant species with disease progression, noting a decrease in *C. albicans* but an increase in *Candida dubliniensis* in advanced stages^16^. This suggests a dynamic interaction between specific fungi and host disease state that may require further validation in a larger cohort. These findings collectively suggest that ALD is associated with a unique intestinal fungal dysbiosis characterized by decreased beneficial fungi, and increased harmful fungi, suggesting that the fungal community may promote ALD progression^44^.

**Table 1:** Mycobiome changes in ALD

Participants	Study type	Genus	Species	Mechanism or conclusion	Detection methods	Ref.
Alcohol-associated cirrhosis (*n* = 4), AH (*n* = 6), AUD (*n* = 10) vs. controls (*n* = 8)	human	*↑Candida* *↓Epicoccum* *↓Debaryomyces*		β-glucan binds to CLEC7A on kupffer cells, inducing hepatitis to promote ALD, leading to steatosis and cell death of hepatocytes.	ITS1 sequencing	^16^
AUD combined with ALD (*n* = 36) vs. controls (*n* = 33)	human		↑*C. albicans*	*C. albicans* specific Th17 cells stimulate the secretion of IL-17, activating the IL17RA signaling pathway on Kupffer cells.	ITS2 sequencing	^43^
AH (*n* = 91), AUD (*n* = 42) vs. controls (*n* = 11)	human		↑*C. albicans*	Candidalysin increases hepatic levels of *Il1b, Cxcl1* and *Cxcl2* mRNAs in mice following ethanol administration. Pro-inflammatory cytokines directly induce hepatocyte death without altering intestinal barrier function.	qPCR	^17^
AH (*n* = 59), AUD (*n* = 15) vs. controls (*n* = 11)	human	↑*Candida* ↓*Penicilllium* ↓*Saccharomyces* ↓*Debaryomyces*		Higher serum ASCA was associated with increased mortality in patients with AH.	ITS1 sequencing	^14^
AUD (*n* = 66) vs. controls (*n* = 18)	human	↑*Candida* ↑*Debaryomyces* ↑*Pichia* ↑*Kluyveromyces* ↑*Issatchenkia* ↑*Scopulariopsis* ↓*Aspergillus*	↑*C. albicans* ↑*Candida zeylanoides* ↑*Issatchenkia orientalis* (synonymous with *Pichia kudriavzevii* and *Candida krusei*) ↑*Scopulariopsis cordiae* ↓*Kazachstania humilis*	1. Improved liver health in AUD patients after abstinence is associated with reduced gut abundance of *Candida* and *Malassezia* and reduced serum IgG levels against *C. albicans;* *2. Malassezia* abundance can distinguish between progressive and non-progressive liver disease in AUD;	ITS2 sequencing	^45^
Active AUD *vs*. abstinent AUD (*n* = 56, paired)	human	↑*Candida* ↑*Malassezia* ↑*Hanseniaspora* ↑*Kluyveromyces* ↑*Cyberlindneria* ↑*Pichia* ↑*Issatchenkia* ↑*Claviceps* ↓*Trichosporon*	↑*C. albicans* ↑*Candida zeylanoides* ↑ *M. restricta* ↑*Cyberlindneria jadinii* ↑*Issatchenkia orientalis*			

*ALD* alcohol-related liver disease, *AUD* alcohol use disorder, *AH* alcoholic hepatitis, *CLEC7A* C-type lectin domain containing 7 A, *ASCA* anti-Saccharomyces cerevisiae antibody, *IgG* immunoglobulin G, *ITS1/2* internal transcribed spacer 1/2, *qPCR* quantitative polymerase chain reaction; *C. albicans Candida albicans, M. restricta Malassezia restricta*.

### Fungi are associated with the occurrence and progression of ALD

Intestinal fungal dysbiosis plays a significant role in the development and progression of ALD. Specific fungal species, such as *C. albicans*, have been closely linked to disease severity and outcomes. For instance, increased abundance of *C. albicans* predicts mortality in patients with alcoholic hepatitis (AH) and correlates with clinical disease scores^14^. The abundance of *Malassezia* is significantly higher in patients with progressive liver disease and decreases after alcohol cessation, highlighting its potential role in disease progression^45^. Beyond mere colonization, fungal components and metabolites also drive the pathogenesis. Fungal cell wall component β-glucan induces IL-1β release by binding to C-type lectin domain containing 7 A (CLEC7A) on Kupffer cells, promoting hepatitis, steatosis, and hepatocyte death, thereby aggravating ALD^16^. Additionally, *C. albicans* secretes a cytolytic peptide toxin known as candidalysin, which is closely related to the severity and mortality of AH. Unlike β-glucan, candidalysin directly induces hepatocyte death without relying on CLEC7A activation or intestinal barrier disruption^17^. The host’s systemic immune response to fungi also serves as a critical biomarker. Elevated anti-*S. cerevisiae* antibodies (ASCA) are observed in AH patients and correlate with increased intestinal permeability, enhanced systemic fungal immunity, and higher mortality^14^. Similarly, anti-*C. albicans* IgG levels reflect disease activity and decrease with alcohol abstinence, paralleling improvements in liver health^45^. Furthermore, *C. albicans* stimulates a specific Th17 cell response, which secretes IL-17 to activate the IL-17RA signaling pathway on Kupffer cells, further promoting ALD development^43^. In conclusion, intestinal fungal dysregulation, mycobiome-derived metabolites, and the host’s antifungal immune response are key factors in the severity and prognosis of ALD, providing promising targets for future antifungal or immunomodulatory treatments.

### Metabolic dysfunction-associated steatotic liver disease (MASLD)

MASLD is the most prevalent CLD globally, with a global prevalence of approximately 32% (estimated in 2022) and is strongly associated with obesity, dyslipidemia, and insulin resistance^46,47^. In China, MASLD affects approximately 29.6% of adults, with a higher prevalence in males (34.8%) than in females (23.5%)^48^. The disease spectrum ranges from simple fatty liver (SFL) and metabolic dysfunction-associated steatohepatitis (MASH) to cirrhosis and hepatocellular carcinoma (HCC)^10,49^, with about 20% of patients progressing to advanced liver disease^50,51^. Emerging studies indicate that alterations in the fecal fungal microbiota are associated with hepatic inflammation and histopathological features such as steatosis and ballooning^15^.

### Changes in the fungal communities of patients with MASLD

Emerging evidence indicates that MASLD is associated with significant alterations in the gut mycobiota composition across multiple taxonomic levels (Table 2). At the phylum level, patients show a significant increase in Ascomycota and a marked decrease in Basidiomycota compared to healthy controls^15,52^. Importantly, a distinct fungal signature has been identified that differentiates MASLD from ALD, highlighting the disease-specific nature of mycobiota dysbiosis^53^. Furthermore, in a recent review by Scarlata et al., they discussed the changes in the gut microbiota of MASLD and inflammatory bowel disease, including fungal dysbiosis, and emphasized the common pathogenic mechanisms of the diseases^54^. These findings collectively indicate that MASLD is associated with specific alterations in intestinal fungi. The disease specificity of these fungal microbiota changes indicates their potential utility as diagnostic biomarkers and therapeutic targets.

**Table 2:** Mycobiome changes in MASLD

Participants	Study type	Genus	Species	Mechanism or conclusion	Detection methods	Ref.
MASH (*n* = 54) vs. MASLD (*n* = 24)	human		↑*C. albicans* ↑*Pichia barkeri* ↑*Mucor* species ↑*Cyberlindneria jadinii* ↓*Malassezia* species	1. The more severe the MASLD, the more *C. albicans*; 2. *Mucor* species were positively associated with MASH, fibrosis and particularly hepatic inflammatory changes;	ITS2 sequencing	^15^
MASH (*n* = 54) vs. control (*n* = 16)			↑log ratio *Babjeviella inositovora*/*S. cerevisiae* ↑log ratio *Mucor* species/*S. cerevisiae*			
Fibrosis stages F2-F4 (*n* = 38) *vs*. F0-F1 (*n* = 40)			↑*C. albicans* ↑*Pichia barkeri* ↑*Mucor* species ↑*Cyberlindneria jadinii* ↓*Penicillium* species ↓*Blumeria* species			
Fibrosis stages F2-F4 (*n* = 38) vs. control (*n* = 16)			↑log ratio *Babjeviella inositovora*/ *S. cerevisiae* ↑log ratio *Mucor* species/*S. cerevisiae*			
MASLD with fibrosis stages F3-F4 (*n* = 24) vs. AUD with F3-F4 (*n* = 11)			↑*Mucor* species ↓*C. albicans* ↓*Candida* species ↓*Debaryomyces* species ↓*Blumeria* species			
MASLD (*n* = 79) vs. control (*n* = 34)	human	↑*Talaromyces* ↑*Paraphaeosphaeria* ↑*Lycoperdon* ↑*Curvularia* ↑*Phialemoniopsis* ↑*Paraboeremia* ↑*Sarcinomyces* ↑*Cladophialophora* ↑*Sordaria* ↓*Leptosphaeria* ↓*Pseudopithomyces* ↓*Fusicolla*		1. The relative abundance of *Cladosporium* fungi was increased in both patients with MASH and those with significant fibrosis; 2.*Lycoperdon*, *Curvularia*, *Cladophialophora*, and *Sordaria* fungi were positively correlated with the clinical variables ALT, AST, and GGT; 3.*Pulvinula* fungi, which were depleted in patients with significant fibrosis, were simultaneously negatively correlated with ALT, AST, and fibrosis stage;	ITS2 sequencing	^52^
MASH (*n* = 15) vs. MASLD (*n* = 17)		*↑Paramycosphaerella* *↑Fusicolla* *↑Arthrinium* *↑Triparticalcar* *↑Trichoderma* *↑Cladosporium*				
Fibrosis stages F2-F4 (*n* = 10) *vs*. F0-F1 (*n* = 22)		↑*Cladosporium* ↑*Staphylotrichum* ↑*Paecilomyces* ↑*Thermomyces* ↓*Pulvinula*				
ALD (*n* = 58), MASLD (*n* = 78) vs. controls (*n* = 34)	human	↑*Saccharomyces* ↑*Kluyveromyces* ↑*Scopulariopsis* ↓*Kazachstania* ↓*Mucor* (in MASLD)	↑*C. albicans* ↑ *M. restricta* ↑*Scopulariopsis cordiae* ↓*Candida argentea* ↓*Pichia kluyveri* (in MASLD)	1. The relative abundance of *C. albicans* correlated with disease severity in both ALD and MASLD; 2.Fungal signature (*Scopulariopsis*, *Kluyveromyces*, *Mucor*, *M.restricta*, *Kazachstania*) differentiates early-stage ALD from MASLD; 3.Fungal signature (*Scopulariopsis*, *Kluyveromyces*, *Mucor*, *M. restricta*) achieves excellent discriminative ability for advanced fibrosis;	ITS2 sequencing	^53^
ALD F0-F1 (*n* = 48) vs. MASLD F0-F1 (*n* = 43)		↑*Kluyveromyces* ↑*Scopulariopsis* ↓*Mucor* (in MASLD)	↑*C. albicans* ↑*M. restricta* ↑*Scopulariopsis cordiae* ↓*Candida argentea* (in MASLD)			
ALD F2-F4 (*n* = 10) vs. MASLD F2-F4 (*n* = 30)		↑*Debaryomyces* ↑*Scopulariopsis* ↑*Kluyveromyces* ↓*Mucor* (in MASLD)	↑*M. restricta* ↑*Scopulariopsis cordiae*			

*ALD* alcohol-related liver disease, *MASLD* metabolic dysfunction-associated steatotic liver disease, *MASH* metabolic dysfunction-associated steatohepatitis, *ALT* alanine aminotransferase, *AST* aspartate aminotransferase, *GGT* gamma-glutamyl transferase, *ITS2* internal transcribed spacer 2, *C. albicans Candida albicans, S. cerevisiae Saccharomyces cerevisiae, M. restricta Malassezia restricta*.

### Fungi are associated with the occurrence and progression of MASLD

Fungal dysbiosis is significantly associated with MASLD progression. A more severe clinical condition (such as with advanced fibrosis) is correlated with higher abundance of *C. albicans* and elevated plasma levels of *C. albicans* IgG. Furthermore, *C. albicans*, *Mucor sp., Cyberlindnera jadinii, Penicillium*, unidentified *Polyporales, Babjeviella inositovora*, and *Candida argentea* are associated with the presence of MASH. In particular, *Mucor sp*. shows a positive correlation with MASH, fibrosis, and especially hepatic inflammation^15^. In contrast, the symbiotic fungus *Fusarium foetens* ameliorates MASH via its metabolite FF-C1-mediated CerS6 inhibition, reducing ceramides and improving steatosis, inflammation, and fibrosis^55^. In addition, the probiotic *Saccharomyces boulardii* (*S. boulardii*) alleviated MASLD by regulating the ratio of *Escherichia coli/Lactobacillus acidophilus* in the intestinal tract of MASLD model mice, reducing body weight, improving hepatic steatosis, and reducing endotoxemia^56^. These findings underscore the dual role of fungi in MASLD: pathogenic fungi like *C. albicans* and *Mucor sp*. exacerbate disease, while beneficial fungi such as *Fusarium foetens* and *S. boulardii* confer protection through metabolite-mediated and immunomodulatory mechanisms. Targeting specific fungi or fungal metabolites may offer novel therapeutic strategies for MASLD.

### Primary sclerosing cholangitis (PSC)

PSC is a chronic autoimmune liver disease. A recent meta-analysis indicates that its incidence and prevalence are 0.87 per 100,000 people and 13.53, respectively^57^. A latest study predicts that the global prevalence of PSC will rise to 22.98 cases per 100,000 people^58^. It is characterized by progressive biliary inflammation and fibrosis, often leading to cirrhosis, portal hypertension, and liver failure, with liver transplantation remaining the only life-prolonging treatment for advanced cases^59,60^. Although its pathogenesis remains unclear, over 80% of PSC patients have comorbid inflammatory bowel disease, suggesting shared immune dysfunction^61,62^. When the immune system weakens, fungal community imbalance occurs^10^. Although most studies have focused on bacterial changes such as increased *Streptococcus* and *Veillonella* and decreased *Faecalibacterium*^63–66^, fungal involvement is supported by clinical observations of severe fungal infections in PSC patients^67,68^ and serological evidence such as elevated ASCA in PSC populations^69^.

### Changes in the fungal communities of PSC patients

Current evidence indicates significant alterations in the gut mycobiome of patients with PSC (Table 3). At the species level, one study reported a specific increase in *Trichocladium griseum* in PSC patients^70^, while another identified *C. albicans* and *Candida glabrata* (the current classification has been updated to *Nakaseomyces glabratus*^71^) in bile duct samples from PSC patients^72^. This fungal dysbiosis is clinically significant, as biliary *Candida* infections are associated with a more severe disease course^72,73^. In summary, PSC patients demonstrate distinct changes in gut fungal composition, particularly increases in *Candida* species and *Trichocladium griseum*, along with a decrease in *Saccharomyces*, suggesting that fungal dysbiosis may be associated with PSC pathogenesis.

**Table 3:** Mycobiome changes in PSC

Participants	Study type	Genus	Species	Mechanism or conclusion	Detection methods	Ref.
PSC (*n* = 33) vs. controls (*n* = 66)	human	↑*Candida*	↑*Trichocladium griseum*	1.*Candida* and *Trichocladium griseum* are consistently elevated in PSC across geographically distinct cohorts; 2.*Candida* may drive Th17 immune responses, linked to PSC pathogenesis;	ITS2 sequencing	^189^
PSC (*n* = 22) vs. controls (*n* = 30)	human	↑*Exophiala* ↓*Saccharomyces*	↓*S. cerevisiae*	1.*Exophiala* (linked to systemic infections) is enriched in PSC, suggesting a potential pathogenic role. 2.Reduced *S. cerevisiae* (anti-inflammatory) may exacerbate inflammation.	ITS2 sequencing	^190^

*PSC* primary sclerosing cholangitis, *ITS2* internal transcribed spacer 2, *S. cerevisiae Saccharomyces cerevisiae*.

### Fungi are associated with the occurrence and progression of PSC

*C. albicans* is the main fungal inducer of the antifungal response in human Th17 cells^74^. Th17 cells appear to play a role in PSC because after stimulating peripheral blood mononuclear cells (PBMCs) with *C. albicans*, the production of Th17 cells in PSC-PBMCs was significantly higher than that in PBMCs of the healthy control group^75^. Th17 cells also aggravate biliary tract inflammation in PSC^76,77^. Patients with PSC accompanied by biliary *candida* infection (most commonly *C. albicans*) have more severe cholangitis and higher levels of C-reactive protein (CRP) and serum bilirubin than those without *candida* infection^72^. Moreover, *Candida* species identified in bile (including *C. albicans, Candida glabrata, Candida tropicalis*, and unidentified *Candida* species) are associated with a poor prognosis in patients with PSC, and these patients require liver transplantation relatively earlier^73^. In summary, *C. albicans* infection and the pathogenic Th17 response it drives are the key factors that exacerbate the severity of PSC and deteriorate clinical outcomes.

### Liver cirrhosis

Cirrhosis is the advanced stage of liver fibrosis caused by long-term liver injury and is the main cause of death in CLDs (such as ALD, viral hepatitis, and MASLD)^70^. During the occurrence and development of cirrhosis, the microbiota, including bacteria (bacterial group), fungi (fungal group), and viruses (viral group), undergoes changes^12^. As the condition worsens, such as in decompensated liver cirrhosis, microbiota dysbiosis is maintained and further exacerbated^78,79^. The main cause of death was intestinal infection^80–82^. Although infections are primarily related to intestinal bacteria, the role of fungi is receiving increasing attention.

### Changes in the fungal communities of patients with liver cirrhosis

Patients with liver cirrhosis exhibit significant alterations in gut fungal composition (Table 4). At the phylum level, a marked increase in Ascomycota and a concomitant decrease in Basidiomycota have been observed^83,84^. Studies report elevated abundances of *Candida glabrata* in cirrhotic patients^83,85^. Pathogenic species including *Cryptococcus neoformans* emerged in spontaneous fungal peritonitis^86,87^. In summary, liver cirrhosis is associated with a distinct shift in fungal ecology characterized by an expansion of Ascomycota and *Candida* species (*C. albicans* and *Candida glabrata*) at the expense of Basidiomycota and *Saccharomyces*.

**Table 4:** Mycobiome changes in liver cirrhosis

Participants	Study type	Genus	Species	Mechanism or conclusion	Detection methods	Ref.
Liver cirrhosis (*n* = 143) vs. controls (*n* = 26)	human	↑*Candida* ↓*Saccharomyces*	Phylum: ↑Ascomycota ↓Basidiomycota	Bacteroidetes/Ascomycota ratio predicts 90-day hospitalizations (↓ratio=higher risk).	ITS sequencing	^83^
Hepatitis B cirrhosis (*n* = 38), chronic hepatitis B (*n* = 35), HBV carriers (*n* = 33), controls (*n* = 55)	human	↑*Candida*	↑*S. cerevisiae*	Fungal diversity positively correlates with disease progression of chronic HBV infection (cirrhosis > chronic hepatitis B > HBV carriers ≈ healthy volunteers)	Culture	^85^

*HBV* hepatitis B virus, *ITS* internal transcribed spacer, *S. cerevisiae Saccharomyces cerevisiae*.

### Fungi are associated with the occurrence and progression of liver cirrhosis

Patients with liver cirrhosis are often colonized by fungi, and the risk of fungal infection is very high^82,86,88^. This is mainly caused by *Candida* and is usually associated with delayed diagnosis, a high incidence of acute exacerbation of chronic liver failure, prolonged hospital stay, admission to the intensive care unit, and a worse 30 day survival rate compared to no infection or bacterial infection^81,89^. Beyond *Candida*, the genera *Cladosporium* and *Paecilomyces* were significantly positively correlated with the fibrosis stage, whereas *Pulvinula*, which was decreased in patients with significant fibrosis, was negatively correlated with the fibrosis stage, ALT, and AST^52^. When liver fibrosis persists, it can gradually progress to liver cirrhosis. Fungi, especially *Candida* spp., have been identified as potential contributors to poor outcomes in pre-cirrhotic and cirrhotic liver disease^83^. According to research, among patients with liver cirrhosis, the prevalence of systemic antibodies against *C. albicans* and *S. cerevisiae* is 24%-59%, and the mortality rate among these patients is very high^90^. In advanced liver diseases, immune dysregulation occurs, and spontaneous fungal peritonitis is mainly caused by *C. albicans*, which is a serious complication of liver cirrhosis and has a poor prognosis^91,92^. Therefore, changes in fungal communities are not only a consequence of the progression of liver cirrhosis but also a key player in driving poor prognosis, especially when *Candida* plays a core role in infection and immune disorders.

### HCC

HCC is a leading cause of global cancer mortality, often associated with CLD and characterized by late diagnosis, high recurrence, and poor survival^93–96^. These challenges highlight the urgent need for novel diagnostic and prognostic markers^97,98^. Current research on the gut mycobiome in HCC remains limited, though aflatoxin, produced by *Aspergillus* species, is an established fungal-derived carcinogen contributing to its pathogenesis^99^.

### Changes in the fungal communities of HCC patients

Patients with HCC exhibit distinct alterations in gut fungal composition (Table 5). At the phylum level, the progression is characterized by an initial increase in Chytridiomycota and subsequent replacement by Ascomycota^100^. Notably, *C. albicans* abundance is higher in patients with advanced TNM stages (III-IV) compared to those with early stages (I-II)^101^. In summary, HCC is associated with a significant restructuring of the gut mycobiota, characterized by an expansion of Ascomycota*, Candida*, and *Malassezia* at the expense of beneficial fungi such as *Saccharomyces*, suggesting that fungal dysbiosis may be implicated in HCC pathogenesis.

**Table 5:** Mycobiome changes in HCC

Participants	Study type	Genus	Species	Mechanism or conclusion	Detection methods	Ref.
HCC (*n* = 17) vs. cirrhosis (*n* = 11)	human	*↑Candida* *↓Kazachstania* *↓Debaryomyces* *↓Xeromyces* *↓Amorphotheca* *↓Blastobotrys*	↑*C. albicans*	Intestinal *C. albicans* promotes HCC by up-regulating NLRP6	ITS1 sequencing	^102^
Mouse model: HCC (*n* = 10) Human samples: HCC (*n* = 6)	human and animal	Phylum: ↑Ascomycota	Mouse: *↑Kazachstania pintolopesii* ↓ *S. cerevisiae* *↓Aspergillus heterocaryoticus*. Human: *↑C. albicans* ↓ *S. cerevisiae*	*C. albicans* promotes HCC progression, while *S. cerevisiae* retards it.	ITS1 sequencing	^100^
HCC (*n* = 34) vs. cirrhotic patients (*n* = 20), controls (*n* = 18)	human	HCC vs. controls: ↑*Candida* ↑*Malassezia* ↑*Rhizopus* ↑*Neocatemulostroma* ↓*Actinomucor* ↓*Mucor* ↓*Alternaria* ↓*Trichocladium* HCC vs. cirrhosis: *↑Candida* *↑Monographella* *↑Bipolaris* *↑Nakaseomyces* *↑Malassezia* *↑Sporothrix* *↑Staphylotrichum* *↓Archaeorhizomyces*	HCC vs. controls: ↑*C. albicans* ↑*Fusarium proliferatum* ↓*Actinomucor elegans* ↓*Mucor circinelloides* ↓*Alternaria alternata*, ↓*Piptoporus mandshuricus* HCC vs. cirrhosis: *↑C. albicans* *↑Candida tropicalis* *↑Monographella nivalis* *↑Sporothrix ramosissima* *↑Staphylotrichum coccosporum* *↓Archaeorhizomyces sp*.	1.*C. albicans* abundance rises with advanced TNM stages (III–IV). 2.*C. albicans* and *Malassezia* can promote HCC tumor growth.	ITS2 sequencing	^101^

*HCC* hepatocellular carcinoma, *NLRP6* NLR family pyrin domain containing 6, *TNM* tumor node metastasis, *ITS1/2* internal transcribed spacer 1/2, *C. albicans Candida albicans, S. cerevisiae Saccharomyces cerevisiae*.

### Fungi are associated with the occurrence and progression of HCC

Although patients with cirrhosis already have elevated fecal levels of *Candida*, patients with HCC have been found to have even higher faecal proportions of *Candida* and *C. albicans* than patients with cirrhosis, but lower proportions of the genera *Kazachstania, Debaryomyces, Xeromyces, Amorphotheca*, and *Blastobotrys*^83,102^. The increased abundance of *C. albicans* and the depletion of *S. cerevisiae* might be markers of liver cirrhosis progressing to early HCC^100^. Supplementing *C. albicans* and *S. cerevisiae* in the diet during liver cirrhosis-HCC progression can either accelerate or delay HCC development. Thus, gut fungi may serve as biomarkers for HCC progression and potential targets for preventive or therapeutic interventions.

In summary, we note that the causal relationship between the gut microbiota and CLDs is the core challenge of current research. On one hand, CLDs themselves may promote fungal dysbiosis^40^. For example, in ALD, the toxicity of alcohol metabolism, along with resulting systemic inflammation, oxidative stress (OxS), and BA metabolism, can directly damage the intestinal epithelium and lead to local immune imbalance, thereby creating conditions that favor the proliferation of fungi such as *C. albicans*^14^. At this point, specific fungal dysbiosis may be considered a consequence of disease progression. On the other hand, an increasing amount of evidence supports that specific fungi play an etiological role in aggravating liver injury^16,103,104^. For instance, administration of *C. albicans* or its secreted toxin, candidalysin, in mice has been shown to directly induce hepatocyte death, thereby aggravating ALD^17^. Meanwhile, the intervention of specific fungi (e.g., *S. boulardii*) can significantly improve liver injury indicators^105,106^. Overall, initial liver injury and intestinal barrier disruption cause fungal dysregulation, and the dysregulated fungal community (especially excessive proliferation of harmful strains) feeds back and accelerates CLDs progression through mechanisms such as metabolites, receptor pathways, and immunomodulation via the gut-liver axis^5,43^. Hence, intestinal fungi and CLDs exhibit a bidirectional causal relationship.

Although the association between intestinal fungal dysbiosis and the severity of various CLDs is increasingly recognized, the limitations of some confounding factors still need to be taken into account. Key confounding factors (such as long-term antibiotic use, dietary habits, continuous alcohol intake, concomitant medications, and underlying immune status) are not adequately recorded or controlled in many studies. These factors can significantly alter the structure of fungal communities, thereby interfering with the judgment of the specific associations between fungi and the disease. Furthermore, different studies employ varied sequencing methods (e.g., ITS1, ITS2, or 18S rRNA) and rely on fungal reference databases (e.g., UNITE, ITSoneDB) that are continuously updated^107,108^, which may introduce biases in species classification, lead to underdetection of low-abundance taxa, and complicate direct comparisons across studies.

Therefore, future research should incorporate detailed clinical data and adopt more longitudinal cohort designs to assess changes in fungal communities over time and their temporal relationship with disease progression. This is essential for clarifying the causal role and mechanisms of specific fungi in the pathogenesis of chronic liver diseases.

## Possible mechanisms by which intestinal fungi regulate the progression of CLDs

### Fungal imbalance and mucosal barrier disruption

Some fungal populations (including *C. albicans*) can be commensal when the host is healthy, but they can invade and cause infection when the intestinal barrier function is impaired and/or the host’s immune function is weakened^109^. This impairment often involves downregulation of key tight junction proteins (e.g., occludin, claudins, zonula occludens-1 [ZO-1]) and mucin components at both RNA and protein levels, which disrupts epithelial integrity and facilitates fungal translocation^19,109^. For example, *Candida tropicalis* can increase intestinal permeability by disrupting the organization and function of claudin-1, a core tight junction protein, likely through hyphal invasion^110^. A membrane-free gut-on-chip study revealed that candidalysin disrupts tight junction proteins (ZO-1, occludin) to induce “leaky gut”, and further induces intestinal epithelial cells (IECs) apoptosis and necroptosis, thereby exacerbating barrier breakdown^111^. Conversely, knocking out the gene ECE1 that only encodes candidalysin results in a sharp decline in IECs damage, barrier disruption and penetration ability^112^. This indicates that candidalysin is critical to IECs damage and fungal translocation.

Functionally, impaired intestinal barrier integrity leads to increased translocation of microbe-associated molecular patterns (MAMPs) into the portal circulation^78^. These products (such as β-glucan, prostaglandin F2α (PGF2α), aflatoxin and candidalysin), upon reaching the liver, are recognized by immune cells including dendritic cells (DCs) and T cells, inducing pro-inflammatory cytokines such as IL-17 and driving Th17-mediated responses that contribute to the progression of CLDs^78,113^.

### Receptor pathways

**Fig. 1:**
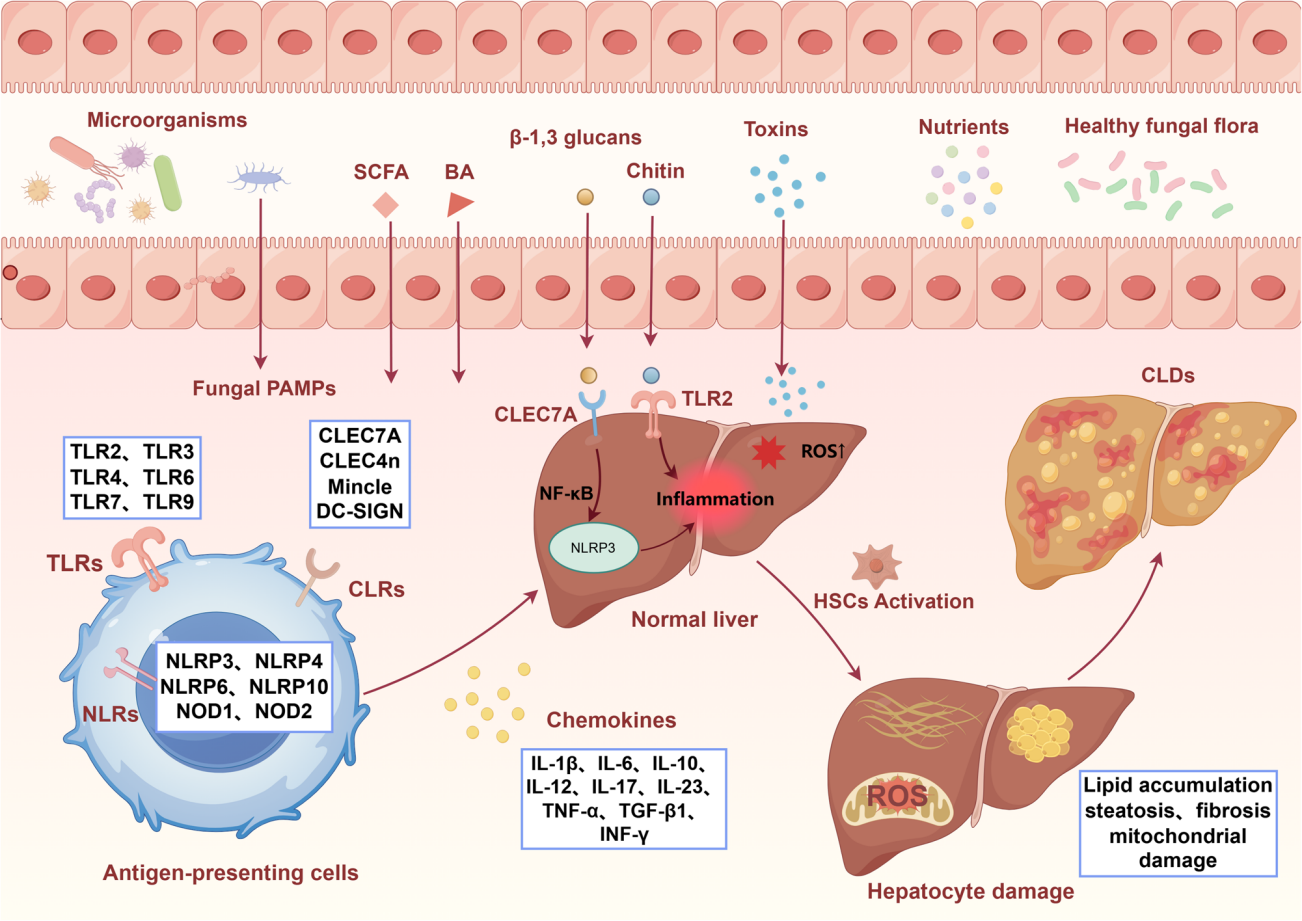
Intestinal fungi regulate chronic liver diseases through receptor pathways. PAMPs carried by healthy fungal microbiota, such as β-1, 3-glucans and chitin, as well as SCFAs, BAs, etc., jointly constitute the intestinal microenvironment. When fungal PAMPs are transported to the intestinal mucosa, they can be recognized by various PRRs on the surface of antigen-presenting cells. It mainly includes: β-1, 3-glucans are recognized by CLEC7A; Chitin is recognized by TLR2, etc. After the receptor is activated, it triggers the downstream NF-κB signaling pathway, assembles the NLRP3 inflammasome, and subsequently releases a large number of pro-inflammatory cytokines (such as IL-1β, IL-6, IL-23, TNF-α, TGF-β1) and chemokines. Inflammatory mediators enter the liver through the portal vein, recruit and activate HSCs. HSCs produce ROS, ultimately lead to liver cell damage, which is manifested as typical characteristics of chronic liver diseases such as lipid accumulation, steatosis, fibrosis and mitochondrial damage. SCFA short-chain fatty acid, BA bile acid, PAMPs pathogen-associated molecular patterns, PRRs pattern recognition receptors, TLRs toll-like receptors, CLRs C-type lectin receptors, NLRs NOD-like receptors, CLEC7A C-type lectin domain containing 7 A, CLEC4n C-type lectin domain family 4, member n, TCR T-cell receptor, IL interleukin, NF-κB nuclear factor kappa-light-chain-enhancer of activated B cells, NLRP3 NLR family pyrin domain containing 3, NOD1/2 nucleotide-binding oligomerization domain-containing protein 1/2, TNF-α tumor necrosis factor alpha, TGF-β1 transforming growth factor beta 1, IFN-γ interferon gamma, ROS reactive oxygen species, HSCs hepatic stellate cells, CLDs chronic liver diseases.

PRRs on immune cells can sense pathogen-associated molecular patterns (PAMPs) on fungi (such as β-glucan and mannan), which induce antigen-presenting cells and initiate an immune response (Fig. 1)^114^. The receptor groups include C-type lectin receptors (CLRs), such as the mannose receptor, CLEC7A, CLEC4n, Mincle, and DC-SIGN; toll-like receptors (TLRs), such as TLR2, TLR3, TLR4, TLR6, TLR,7 and TLR9; nucleotide-binding oligomerization domain-like receptors (NLRs), such as NOD-like receptor protein 3 (NLRP3), NLRP4, NLRP6, NLRP10, nucleotide-binding oligomerization domain-containing protein 1 (NOD1), and 2 (NOD2); and galectin 3^18,114^. Genetic evidence and experimental studies have underlined a central role for CLRs in antifungal immunity, whereas TLRs and NLRs mostly play a secondary role^115,116^.

CLRs (such as CLEC7A) recognize several molecules present in the fungal cell wall (such as β-glucan)^115,116^. β-glucans are glucose polymers that are generally obtained from the cell walls of yeast, such as *Candida*, *Aspergillus*, and *Pneumocystis*^117^. Most pathogenic fungi can release a large amount of β-glucan during their growth in infected hosts. After being recognized by CLEC7A on liver immune cells, the NLRP3 inflammasome is activated, thereby releasing a large amount of inflammatory factors (such as IL-1β) and inducing inflammatory responses^118^. Normal livers are continuously exposed to microbial components derived from the gut microbiota, but no inflammatory response occurs. This is because the TLR signaling pathway functions to maintain tolerance^119^. For example, the pathogenicity of *M. restricta* is related to its cell wall component chitin. Chitin is recognized by TLR2 on the surface of liver immune cells, activating a strong pro-inflammatory response that leads to liver inflammation and damage^104^. Both TLRs and CLRs play a crucial role in the development of liver steatosis, inflammatory damage, and fibrosis.

### Immune pathways

**Fig. 2:**
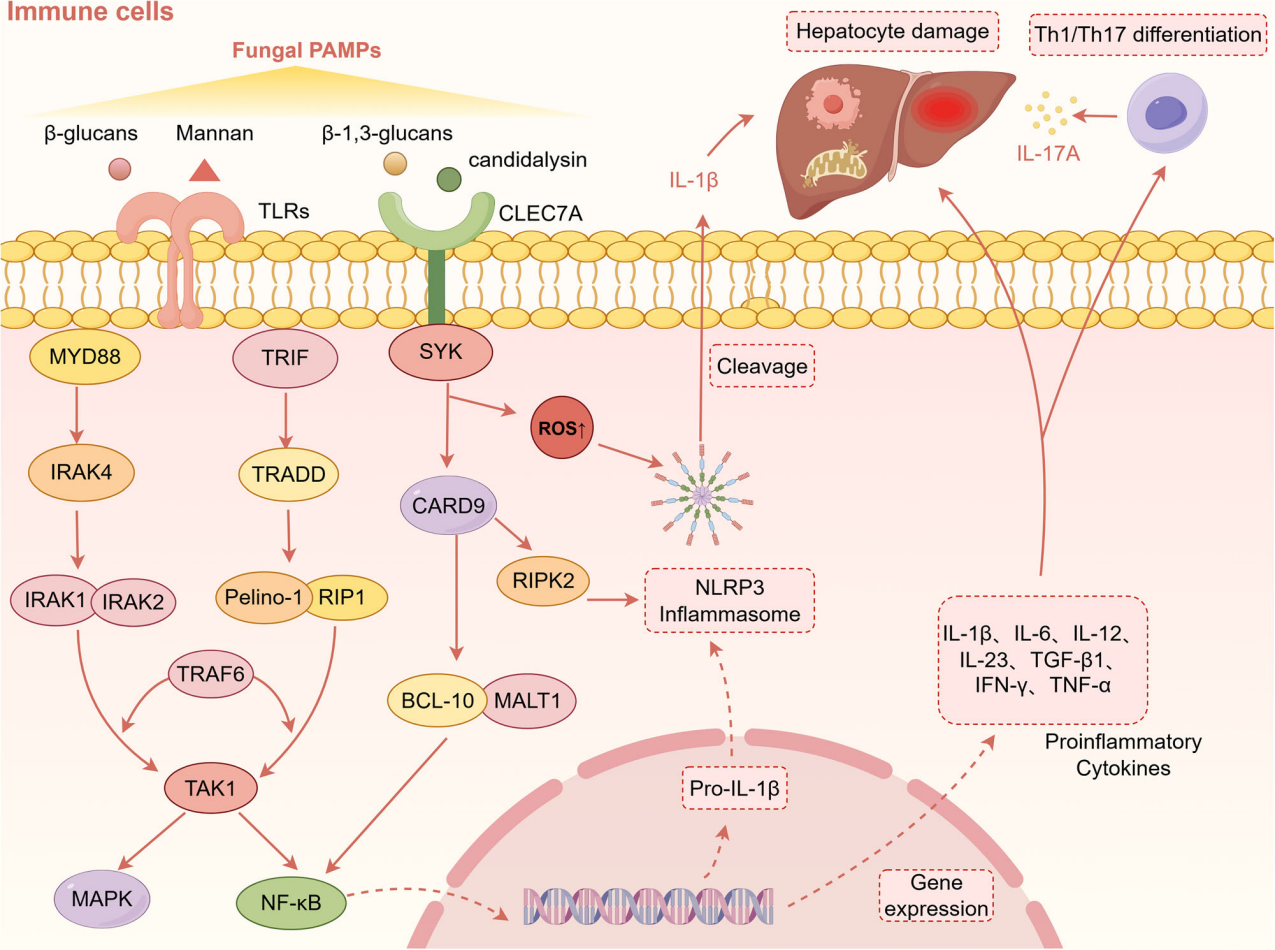
Intestinal fungi regulate chronic liver diseases through immune pathways. Fungal PAMPs, such as β-glucans, β-1,3-glucans, mannan, and candidalysin, are recognized by PRRs on immune cells, including TLRs and CLEC7A. Receptor engagement initiates distinct but interconnected signaling pathways: TLR activation recruits adaptor proteins MYD88 or TRIF. MYD88 recruitment triggers activation of IRAK4, which phosphorylates and activates IRAK1 and IRAK2. Activated IRAK1/2 interacts with TRAF6 to propagate the signal to TAK1. In the TRIF-dependent pathway, TRIF sequentially recruits TRADD and RIPK1. This complex recruits TRAF6. Pelino-1 is recruited to this complex and acts as a critical signal amplifier by catalyzing K63-linked ubiquitination, ensuring robust TAK1 activation. Binding of β-1,3-glucans to CLEC7A activates SYK, which phosphorylates and recruits CARD9, promoting formation of the CARD9–BCL-10–MALT1 (CBM) complex. This pathway synergizes with TLR signaling to enhance NF-κB activation and, through RIPK2, promotes NLRP3 inflammasome assembly. TAK1 serves as a signaling hub, simultaneously activating the NF-κB and MAPK pathways, driving proinflammatory cytokine gene expression. Concurrently, the NLRP3 inflammasome mediates cleavage of pro-IL-1β into its mature, active form (IL-1β). The synergistic output is the release of proinflammatory cytokines (e.g., IL-1β, IL-6, IL-12, IL-23, TGF-β1, IFN-γ, TNF-α). These mediators orchestrate Th1/Th17 cell differentiation and subsequent IL-17A production. Additionally, candidalysin directly activates Th17 responses by promoting IL-1β and IL-23 secretion, further amplifying IL-17A production. Ultimately, these pathways drive hepatocyte damage and contribute to the pathogenesis of chronic liver disease. TLRs toll-like receptors, CLEC7A C-type lectin domain containing 7 A, MYD88 myeloid differentiation primary response 88, TRIF TIR-domain-containing adapter-inducing interferon-β, SYK spleen tyrosine kinase, IRAK interleukin-1 receptor-associated kinase, TRADD TNFR1-associated death domain protein, RIP1 receptor-interacting serine/threonine-protein kinase 1, CARD9 caspase recruitment domain-containing protein 9, RIPK2 receptor-interacting serine/threonine-protein kinase 2, TRAF6 TNF receptor-associated factor 6, TAK1 TGF-β-activated kinase 1, NF-κB nuclear factor kappa-light-chain-enhancer of activated B cells, NLRP3 NLR family pyrin domain containing 3, BCL-10 B-cell lymphoma/leukemia 10, MALT1 mucosa-associated lymphoid tissue lymphoma translocation protein 1, MAPK mitogen-activated protein kinase, ROS reactive oxygen species, PAMPs pathogen-associated molecular patterns, IL interleukin, TGF-β1 transforming growth factor beta 1, IFN-γ interferon gamma, TNF-α tumor necrosis factor alpha, Th T helper cell.

The balanced state maintained by the interaction between intestinal fungi and the immune system is the key to preventing the pathogenicity of symbiotic fungi (Fig. 2). In the ALD mouse model, alcohol disrupts the intestinal barrier, causing translocation of fungi (such as *C. albicans*) and their cell wall component β-glucan^16,120^. Once these receptors recognise a fungus, they trigger signaling cascades (such as the SYK-CARD9, MYD88, TRIF pathways) to produce cytokines, *e.g*., IL-1β, IL-6, IL-12, IL-23, transforming growth factor-β (TGF-β1), and interferon-γ, which induce IL-17A production by Th 17 and Th1 cells^18,121^. Another study on *C. albicans* has shown that candidalysin stimulates the production of IL-1β through a CARD9-dependent mechanism, thereby leading to neutrophil recruitment mediated by the chemokine CXCL1^122^. Activated neutrophils secrete myeloperoxidase (MPO), neutrophil elastase (NE), protease 3, histamine and matrix metalloproteinase 9 (MMP-9), all of which increase OxS, promote liver injury and the proliferation of hepatic stellate cells (HSCs), thereby increasing liver fibrosis^123,124^. Fungi that enter the human body are usually controlled by the innate immune system to prevent the occurrence of diseases. When the immune response is disordered or fails, it leads to pathogen invasion and disease development.

### Metabolites or toxins

Gut fungal metabolites significantly contribute to the progression of CLDs through multiple interconnected mechanisms. Among these, triglycerides, ethanol, prostaglandins (PGs), candidalysin, and aflatoxins play prominent roles. For instance, fungi such as *Candida* species produce triglycerides, which correlate with hepatic lipid accumulation and metabolic dysfunction in MASLD^125,126^. Additionally, several fungi, including *Saccharomyces* and *Candida*, are capable of ethanol production both in vitro and within the gastrointestinal tract, leading to gut fermentation syndrome (GFS), which is frequently complicated by liver cirrhosis^127–129^. Some fungi such as *Candida* and *Pichia kudriavzevii* (*P. kudriavzevii*) (synonymous with *Issatchenkia orientalis* and *Candida krusei*) can produce ethanol by using fructose as a substrate, promoting MASH progression^125^.

Moreover, fungal species such as *C. albicans* and *M. guilliermondii* synthesize prostaglandins, notably prostaglandin E2 (PGE2), which promotes insulin resistance, hepatic steatosis, and inflammation, thereby accelerating MASLD and ALD^130–134^. PGE2 also facilitates Th17-mediated inflammation and fibrosis via IL-17, amplifying liver injury^135–137^. Another key virulence factor is candidalysin, a cytolysin produced by *C. albicans*, which aggravates ALD and activates the NLRP3 inflammasome, a central mediator in MASLD/MASH development^17,138–140^. Furthermore, aflatoxins, especially aflatoxin B1 (AFB1) produced by *Aspergillus* species, induce DNA damage and mutations (e.g., in TP53), vastly increasing HCC risk, particularly in the context of HBV coinfection^141–143^. Collectively, these fungal metabolites and toxins orchestrate a range of pathogenic processes including metabolic dysregulation, inflammation, fibrosis, and carcinogenesis, thereby driving the initiation and progression of various CLDs.

### Interaction between fungi and bacteria

The interactions between intestinal fungi and bacteria, including mutualism, competition, symbiosis, and parasitism, play a crucial role in CLDs progression. For instance, in high-fat diet (HFD) mice, *Talaromyces* was positively associated with *Bifidobacterium*; *C. albicans* was negatively associated with *Clostridium*; and *Penicillium corylophilum* was negatively associated with *Clostridiaceae*^144^. In addition to the microbiota itself, intestinal fungi inhibit bacterial colonization by producing alcohol, antibacterial peptides, and some metabolites^145,146^. For example, the colonization of *C. albicans* in the mouse intestine induces systemic Th17 immune responses^147^, as well as sustained proliferation of bone marrow progenitor cells dependent on IL-6R signaling^148^, thereby protecting the host from invasive *Staphylococcus aureus* infections. In turn, bacteria also regulate fungal development and mycelial growth by producing fatty acids, lactic acid, and butyric acid^149^. A recent study revealed that *Lacticaseibacillus rhamnosus* (formerly *Lactobacillus rhamnosus*) in the gut reduces the pathogenicity of *C. albicans* when it is metabolically active and proliferating. *Lacticaseibacillus rhamnosus* colonization of gut epithelial cells results in the production of specific metabolites, such as phenyllactic acid, hydroxyphenyllactic acid, 2-hydroxyisocaproic acid, and 3-hydroxydecanoic acid, which antagonize *C. albicans*. Furthermore, *Lacticaseibacillus rhamnosus* depletes nutritional sources for *C. albicans*, affecting its metabolism and transcription and forcing *C. albicans* into an unfavorable growth environment, thereby reducing the pathogenicity of *C. albicans*^150^. In addition to *lactobacilli*, other bacterial species including *Pseudomonas* and *Burkholderia*, as well as bacterial metabolites such as butyrate, inhibit *Candida* yeast to hypha transition; a process needed for the formation of resistant biofilms of fungi and bacteria^151,152^. Besides, commensalism is also a common example of interaction between fungi and bacteria. For example, *C. albicans* supports the growth of strict anaerobes such as *Clostridioides difficile* under aerobic conditions^153^. This discovery explains why *C. albicans* colonization significantly reduces the efficacy of fecal microbiota transplantation (FMT) in the treatment of *Clostridioides difficile* infection (CDI).

In conclusion, there exists a complex and inseparable relationship between intestinal bacteria and fungi. Intestinal bacteria have been proven to play an important role in CLDs. Therefore, fungi may affect the progression of CLDs by interacting with the intestinal bacteria. However, there is currently no clear relevant evidence, and the regulatory mechanisms of complex interactions remain unknown.

### Strategies for targeting intestinal fungi in the treatment of CLDs

New therapies based on the fungal community, such as dietary intervention, FMT, probiotics (including fungal and bacterial strains), and antifungal drugs, have shown significant effects in regulating the intestinal fungal community and restoring imbalanced immune homeostasis by re-establishing the core intestinal fungal community^13,154–156^.

### Probiotics

Probiotics, comprising non-pathogenic microorganisms such as *Lactobacillus* and *Bifidobacterium*, demonstrate beneficial effects in CLDs. Among fungal probiotics, *S. boulardii* is the most extensively studied^157^. It ameliorates various forms of experimental liver injury: attenuating drug-induced hepatic lipid peroxidation and glutathione depletion, improving D-galactosamine-induced transaminase levels and hepatocyte necrosis, hemorrhage, inflammatory infiltration, and reducing fibrosis, inflammation, lipid peroxidation, intestinal permeability, and systemic LPS levels in carbon tetrachloride-induced models^106,158,159^. Similarly, kefir, a multi-species probiotic beverage, modulates gut bacterial and fungal communities, suppresses hepatic steatosis, and reduces pro-inflammatory markers in HFD-fed mice^160,161^. However, caution is warranted as fungal probiotics may pose risks of invasive infection in immunocompromised or critically ill individuals^13^.

### Antifungal drugs

Antifungal drugs are a primary therapy for fungal infections, especially in immunocompromised patients, and can be life-saving^162,163^. Common classes include azoles, polyenes, echinocandins, and pyrimidines, such as fluconazole, amphotericin B, and caspofungin, which act by disrupting fungal cell membrane integrity, wall synthesis, or nucleic acid function, thereby reducing fungal colonization and restoring gut microbiota balance^164,165^. Notably, the majority of evidence supporting the therapeutic potential of antifungal agents in CLDs is derived from preclinical models. For instance, in animal studies, amphotericin B reduced alcohol-induced liver injury and steatosis, as well as western diet-induced steatohepatitis^16,52^. Furthermore, treatment with amphotericin B, fluconazole, or 5-fluorocytosine improved MASLD phenotypes, including hepatic steatosis, insulin resistance, and obesity, in HFD-fed mice^166^.

However, targeting specific gut microbiota with antifungals often yields unsatisfactory results, as some drugs are rapidly metabolized and fail to reach the lower digestive tract^167^. While extended delivery may partly help, it can harm commensal microbiota and worsen inflammation^167,168^. Meanwhile, long-term use also disrupts intestinal microbiota (bacteria and fungi), risks multidrug-resistant fungi, and carries hepatotoxic or even fatal side effects^169,170^. Thus, careful consideration of patient groups and inflammatory status is essential when employing prolonged antifungal regimens. Given that the direct evidence from human intervention trials is still limited, further clinical research is needed in the future to rigorously evaluate the safety and efficacy of antifungal drugs in patients with specific CLDs.

### FMT

FMT involves transferring functional microbiota from healthy donors to patients to restore gut microbial balance. Growing evidence suggests FMT holds promise for treating various CLDs, including MASLD, PSC, chronic hepatitis B, and HCC^171–174^. It is important to note that while promising, much of the mechanistic insight and efficacy data originate from animal studies and early-phase human trials. For instance, clinical studies demonstrate that FMT can improve microbiota diversity, reduce complications such as hepatic encephalopathy and ascites, and significantly enhance survival in patients with AH^175–178^. Furthermore, FMT has been shown to ameliorate MASLD, with lean patients often exhibiting better responses than those with obesity^179^. Proposed mechanisms include phage modulation, activation of the BA-FXR-fibroblast growth factor 19 (FGF19) axis, restoration of gut barrier integrity, and enhanced production of beneficial microbial metabolites such as short-chain fatty acids (SCFAs)^180,181^. Therefore, these mechanisms and therapeutic benefits require further validation in large-scale, controlled human trials.

The efficacy of FMT is highly dependent on donor microbiota composition, which varies with geography, diet, and lifestyle^165^. Given the association of *Candida* species with reduced FMT efficacy, administering antifungal agents prior to FMT has been suggested to suppress pathogenic fungi like *C. albicans*, thereby enhancing FMT outcomes^182^. Additionally, modulating recipient immune status may facilitate microbial colonization and improve outcomes^183^. However, FMT is not without risks: it may transmit opportunistic fungi (e.g., *Candida* spp.) and multidrug-resistant bacteria, potentially leading to severe infections, particularly in immunocompromised recipients^165,184^. Therefore, FMT is best applied in early disease stages to evaluate its potential in modifying liver pathology and delaying CLD progression, rather than as a standalone curative approach in advanced cirrhosis^185^.

### Dietary intervention

Diet plays a key role in shaping the gut fungal community. Dietary intake influences fungal abundance and composition. For instance, *Saccharomyces* may originate from yeast-containing foods, while dietary SCFAs exhibit a negative correlation with *Aspergillus* load^186^. An increase in dietary carbohydrate consumption leads to enrichment of *Candida* species. In contrast, diets rich in indigestible carbohydrates (such as dietary fibers) significantly increase the levels of *Bifidobacteria* and *Lactobacilli* in the gut^187,188^. It has been reported that the abundances of the *Alternaria, Saccharomyces, Septoriella*, and *Tilletiopsis* genera were higher in mice administered with normal chow compared with those fed with HFD^144^. Given these effects, dietary modulation through probiotics and prebiotics represents a promising yet underexplored strategy for targeting gut fungi in CLDs.

### Summary and outlook

In recent years, intestinal fungi, as the invisible driver of the progression of CLDs, have seen their research move from the community to a new stage of mechanism analysis and precise intervention. The summary of fungal changes in ALD, MASLD, PSC, liver cirrhosis, and HCC compared with healthy individuals is presented in Table 6. Although sequencing has revealed fungal diversity, mechanistic insights into their pathogenic or protective roles remain limited. Existing evidence indicates that some fungi (such as *C. albicans*) can directly induce liver injury. Understanding the role of fungal components (e.g., β-glucan) and metabolites (e.g., candidalysin) may offer new therapeutic strategies. Current treatments such as probiotics, antifungal drugs, and FMT have shown efficacy and tolerability in clinical trials. However, broader and more precise interventions, such as engineered microbes, postbiotics, or bacteriophage therapy, may be needed to selectively target fungal pathways.

**Table 6:** Fungal changes in ALD, MASLD, PSC, liver cirrhosis, and HCC compared with healthy individuals

Disease type	Genus		Species		Ref.
	Increased	Decreased	Increased	Decreased	
**ALD**	*↑Candida* ↑*Pichia* ↑*Kluyveromyces* ↑*Issatchenkia* ↑*Scopulariopsis*	*↓Epicoccum* ↓*Penicilllium* ↓*Saccharomyces* ↓*Aspergillus*	↑*C. albicans* ↑*Candida zeylanoides* ↑*Issatchenkia orientalis* ↑*Scopulariopsis cordiae*	↓*Kazachstania humilis*	^14,16,17,43,45^
**MASLD**	↑*Talaromyces* ↑*Paraphaeosphaeria* ↑*Lycoperdon* ↑*Curvularia* ↑*Phialemoniopsis* ↑*Paraboeremia* ↑*Sarcinomyces* ↑*Cladophialophora* ↑*Sordaria* ↑*Saccharomyces* ↑*Kluyveromyces* ↑*Scopulariopsis*	↓*Leptosphaeria* ↓*Pseudopithomyces* ↓*Fusicolla* ↓*Kazachstania* ↓*Mucor*	↑*C. albicans* ↑ *M. restricta* ↑*Scopulariopsis cordiae*	↓*Candida argentea* ↓*Pichia kluyveri*	^52,53^
**PSC**	↑*Candida* ↑*Exophiala*	↓*Saccharomyces*	↑*Trichocladium griseum*	↓*S. cerevisiae*	^189,190^
**Liver cirrhosis**	↑*Candida* ↓*Saccharomyces*				^83^
**HCC**	*↑Candida* ↑*Malassezia* ↑*Rhizopus* ↑*Neocatemulostroma* *↑Monographella* *↑Bipolaris* sp. *↑Nakaseomyces* sp. *↑Sporothrix* *↑Staphylotrichum*	↓*Actinomucor* ↓*Mucor* ↓*Alternaria* ↓*Trichocladium*	↑*C. albicans* ↑*Malassezia* sp. ↑*Fusarium proliferatum*	↓*Actinomucor elegans* ↓*Mucor circinelloides* ↓*Alternaria alternata* ↓*Piptoporus mandshuricus*	^101^

*ALD* alcohol-related liver disease, *MASLD* metabolic dysfunction-associated steatotic liver disease, *PSC* primary sclerosing cholangitis, *HCC* hepatocellular carcinoma, *C. albicans Candida albicans*, *S. cerevisiae Saccharomyces cerevisiae*, *M. restricta Malassezia restricta*.

Through this review, we have discovered that various fungi are associated with the progression of liver diseases. Among them, *C. albicans* and its virulence factor candidalysin are considered a promising fungal target with clinical translational potential. This fungus is consistently enriched in various CLDs (including ALD, MASLD, cirrhosis, and HCC) and is associated with disease severity and poor prognosis, suggesting that targeting this single pathogen could have broad therapeutic effects. Furthermore, its cytolytic toxin candidalysin has been shown to directly damage hepatocytes and trigger inflammation, providing a clear molecular target for intervention. For instance, future efforts could focus on developing monoclonal antibodies or small molecule inhibitors targeting candidalysin itself. This approach would enable targeted intervention against the harmful metabolites without disrupting the commensal fungal community, offering a safer treatment strategy compared to broad-spectrum antifungal drugs.

Meanwhile, we also note that although existing studies have established significant associations between intestinal fungi and CLDs, the generalizability of their conclusions is limited by relatively small sample sizes (as shown in Tables 1–5). While small sample studies can provide preliminary, hypothesis-generating evidence, their statistical power is limited and they are susceptible to individual differences, which may lead to biased or imprecise results. Therefore, in order to transform intestinal fungi from promising biomarkers into reliable diagnostic and therapeutic targets, it is recommended that large-scale, multi-center, prospective longitudinal cohort studies be conducted in the future.

Despite progress, key questions remain. Future studies should establish causal links between fungal dysbiosis and liver disease, particularly in conditions like MASLD and ALD, using longitudinal designs. Identifying fungal biomarkers for non-invasive diagnosis and differentiating between diseases like MASLD and ALD also holds promise. Moreover, current research focuses heavily on specific liver diseases, neglecting cross-kingdom interactions among fungi, bacteria, and viruses. Developing new methods to study these complex networks will be essential to unlocking the full therapeutic potential of the gut mycobiota.

## Data Availability

No datasets were generated or analysed during the current study.
